# Prediction of uridine modifications in tRNA sequences

**DOI:** 10.1186/1471-2105-15-326

**Published:** 2014-10-02

**Authors:** Bharat Panwar, Gajendra PS Raghava

**Affiliations:** Bioinformatics Centre, CSIR-Institute of Microbial Technology, Sector 39A, Chandigarh, India; Department of Computational Medicine and Bioinformatics, University of Michigan, Ann Arbor, MI 48109 USA

**Keywords:** Uridine modifications, Pseudouridine, Dihydrouridine, 5-methyl-uridine, tRNAmod

## Abstract

**Background:**

In past number of methods have been developed for predicting post-translational modifications in proteins. In contrast, limited attempt has been made to understand post-transcriptional modifications. Recently it has been shown that tRNA modifications play direct role in the genome structure and codon usage. This study is an attempt to understand kingdom-wise tRNA modifications particularly uridine modifications (UMs), as majority of modifications are uridine-derived.

**Results:**

A three-steps strategy has been applied to develop an efficient method for the prediction of UMs. In the first step, we developed a common prediction model for all the kingdoms using a dataset from MODOMICS-2008. Support Vector Machine (SVM) based prediction models were developed and evaluated by five-fold cross-validation technique. Different approaches were applied and found that a hybrid approach of binary and structural information achieved highest Area under the curve (AUC) of 0.936. In the second step, we used newly added tRNA sequences (as independent dataset) of MODOMICS-2012 for the kingdom-wise prediction performance evaluation of previously developed (in the first step) common model and achieved performances between the AUC of 0.910 to 0.949. In the third and last step, we used different datasets from MODOMICS-2012 for the kingdom-wise individual prediction models development and achieved performances between the AUC of 0.915 to 0.987.

**Conclusions:**

The hybrid approach is efficient not only to predict kingdom-wise modifications but also to classify them into two most prominent UMs: Pseudouridine (Y) and Dihydrouridine (D). A webserver called *tRNAmod* (http://crdd.osdd.net/raghava/trnamod/) has been developed, which predicts UMs from both tRNA sequences and whole genome.

**Electronic supplementary material:**

The online version of this article (doi:10.1186/1471-2105-15-326) contains supplementary material, which is available to authorized users.

## Background

Post-transcriptional modification plays an imperative role in tRNA secondary and tertiary structure formation [1, 2], stability [3–6] and ultimately affects tRNA functions [7, 8]. Sometimes it leads to the alternative folding of tRNAs [1]. It provides structural flexibility to tRNA and rigidifies certain regions to fine-tune the molecule for maximum performance [3, 9]. It affects the gene expressions [10], translation speed and accuracy [11, 12]; enhances the accuracy of codon binding [13] and codon discrimination ability of tRNAs [14]. Modification prevents frame shifting [15, 16] that is required for the maintenance of proper translational reading frame [10, 17] and enables translocation of the tRNA from A to P site [18]. Some modified bases, particularly modifications of anticodon domain help in the amino-acylation reaction of aminoacyl-tRNA synthetases through recognition of cognate tRNAs [19–21]. It is a principal reaction for the precise flow of genetic information into protein sequences [22, 23].

The position of modified base in tRNA sequence is also important because modified wobble position 34 expands tRNA ability to read more than one codons [24]. It contributes 30-40% of all codon recognition depending on the codon usage of an organism [8]. U_34_ is mostly modified [25, 26] and is responsible for the majority of wobble based codon recognitions [7, 8, 27, 28]. The tRNA modifications are involve in various diseases such as Type 2 diabetes [29–31], Cancer [32–35] and mitochondrial disease [36]. Modification also plays important role in human immunodeficiency virus selection of a specific human tRNA to prime reverse transcription [37]. A recent study showed that tRNA modifications play an important role in genome structure and codon usage [38]. Yet, cellular and functional dynamics of tRNA modifications is unexplored and poorly understood due to the absence of large-scale analysis and quantification of modifications. The experimental determination of tRNA modification is also an expensive, tedious and labor-intensive process. Therefore, there is a need to develop an algorithm for the prediction of tRNA modifications.

In this study, an attempt has been made to develop *in-silico* technique for identification of modified bases in tRNA sequence. We retrieved and analyzed modified tRNA from MODOMICS database [39, 40] and observed that most of the modifications are uridine-derived. Therefore, we focused our study on the prediction of uridine modifications (UMs) in tRNA. We used various features such as compositions, binary and structural information of tRNA for developing Support Vector Machines (SVMs) based models for identification of modified uridine in tRNA. It was observed that Pseudouridine (Y) and Dihydrouridine (D) were more prevalent modifications in the tRNA sequences. Therefore separate prediction models have been developed for these (D and Y) prominent uridine modifications (UMs). It is known that modification varies between different kingdoms; therefore we also developed kingdom-specific prediction models. This sequence based prediction and classification of UMs will help the scientific community to explore tRNA biology. In this era of Next-Generation Sequencing (NGS), tRNAmod tool developed in this study will be useful for the genome-wide prediction of tRNA modifications.

## Results

In this study, two different version of MODOMICS database update 2008 [39] and update 2012 [40] have been used. Update 2008 and 2012 of MODOMICS database were containing 218 and 642 tRNA (modified) sequences respectively. In the analysis part, we used all the 642 tRNAs of the 2012 update. We analyzed position-specific base conservation in standard 1–99 (or 0–98) representation using WebLogo [41] and observed that some positions were conserved whereas most of positions have variants (Additional file 1: Figure S1). Thus, determination of position-specific modification in the variable region is a major challenge.

### Analysis of all tRNA modifications

**Figure 1:**
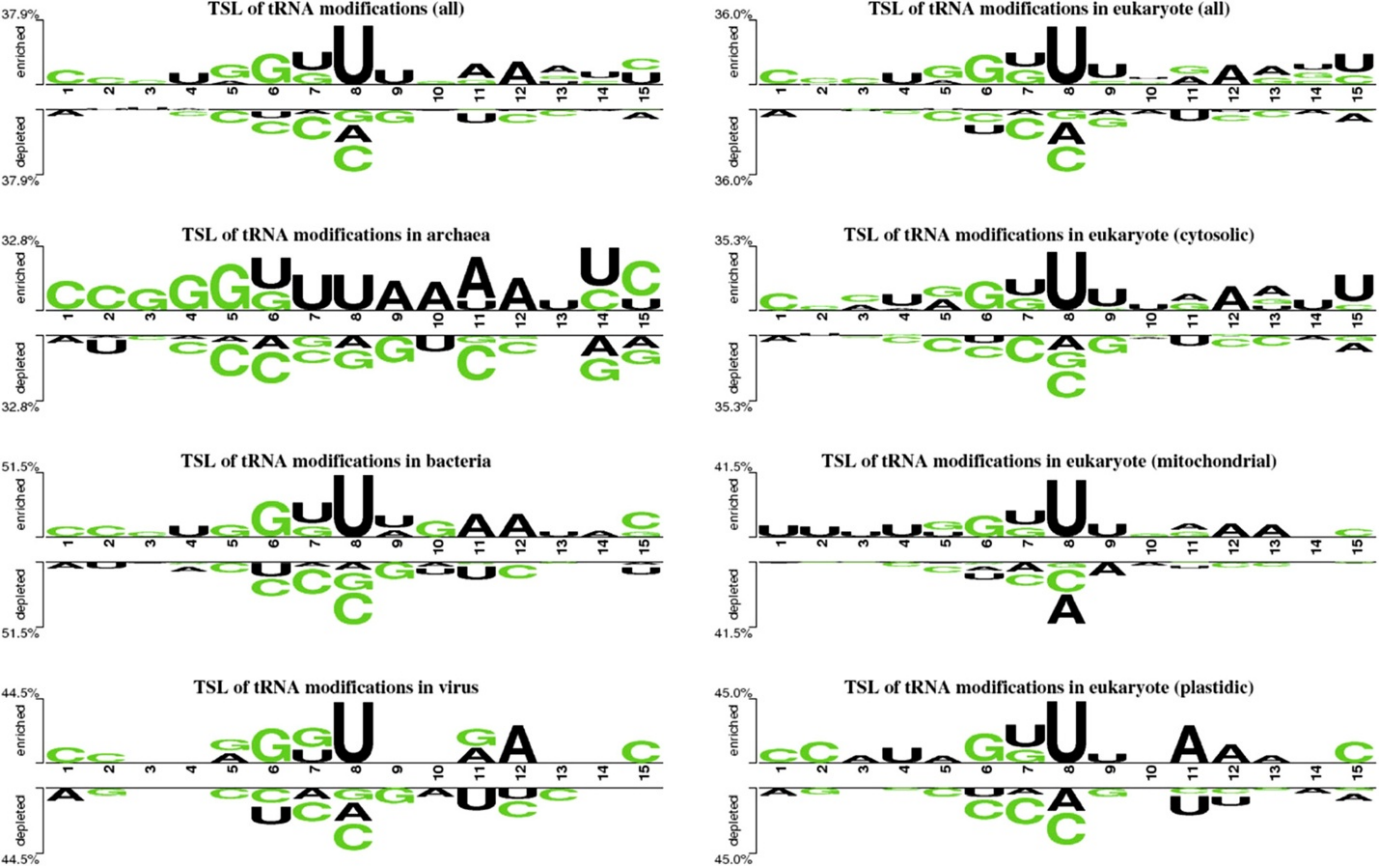
**Kingdom-wise Two-Sample Logos of all modified and unmodified bases using 15-length sliding window patterns.** All bases of central position (8th) are modified and unmodified for positive and negative samples respectively.

We observed that ~13% bases were modified. It means that on an average, 10 bases of each tRNA (average 77 nucleotides long) were modified. The nucleotide-compositions of U, G, A, C and any/other in tRNA sequences were 24.31%, 27.62%, 22.5%, 25.34% and 0.22% respectively. The U-, G-, A- and C-derived modifications were 55.85%, 19.71%, 12.28% and 10.41% of all tRNA modifications respectively. Base specific modification rate varies between the different kingdoms but still uridine-derived modifications are most abundant in all the kingdoms (Additional file 1: Figure S2). The 29.27% of all uridines were modified whereas only 9.09% of guanines, 6.94% of adenines and 5.21% of cytosines were modified. We observed in the kingdom-wise Two Sample Logos (TSLs) of modified and unmodified patterns (sliding windows of 15 lengths) that uridines were also most abundant in the neighboring positions of modified base whereas cytosines and guanines were preferred in the neighboring positions of unmodified bases (Figure 1). Most of modifications were uridine-derived; therefore we selected only these modifications for further study.

### Analysis of uridine modifications (UMs)

**Figure 2:**
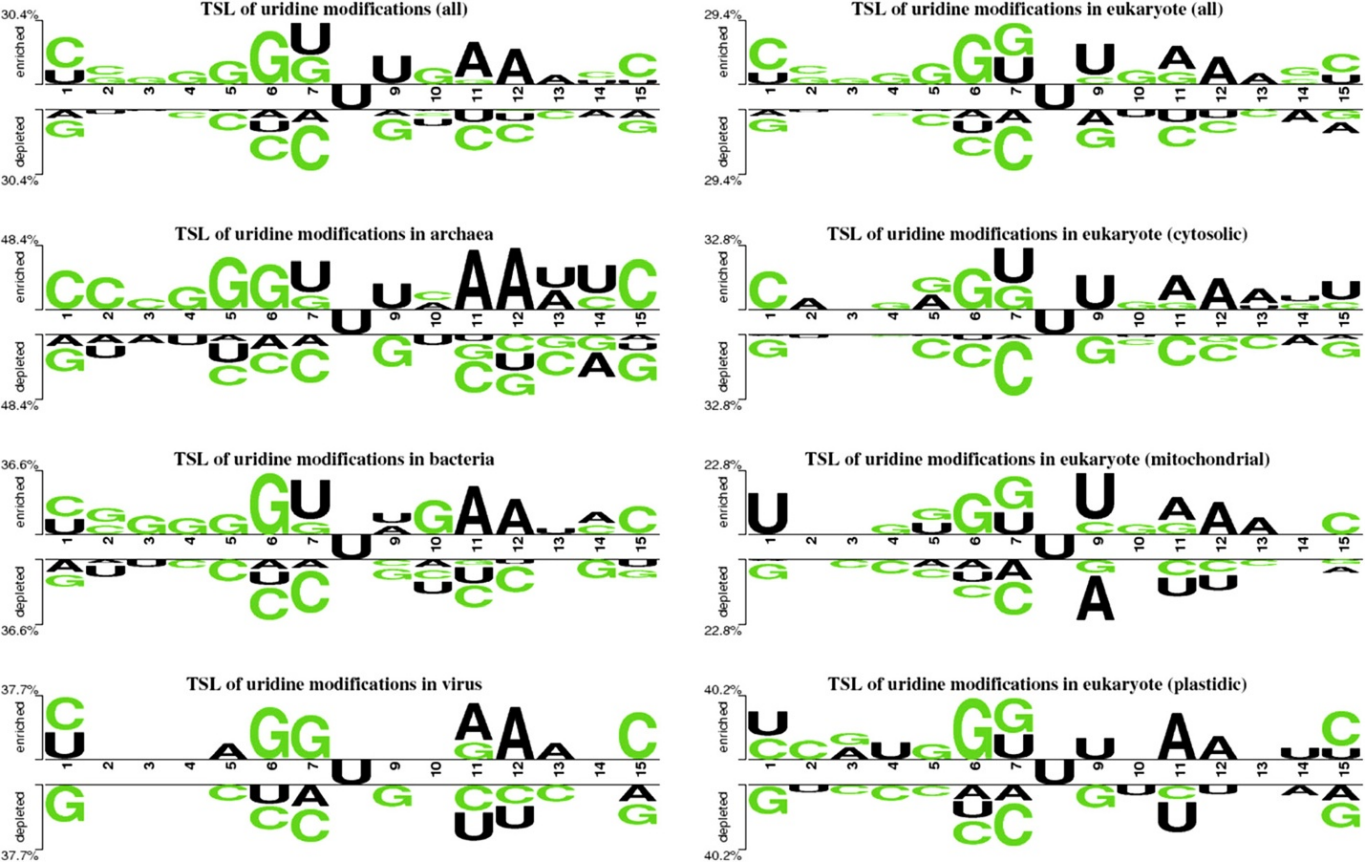
**Kingdom-wise Two-Sample Logos of modified and unmodified uridines using 15-length sliding window patterns (central 8th position for modified/unmodified uridines).**

**Figure 3:**
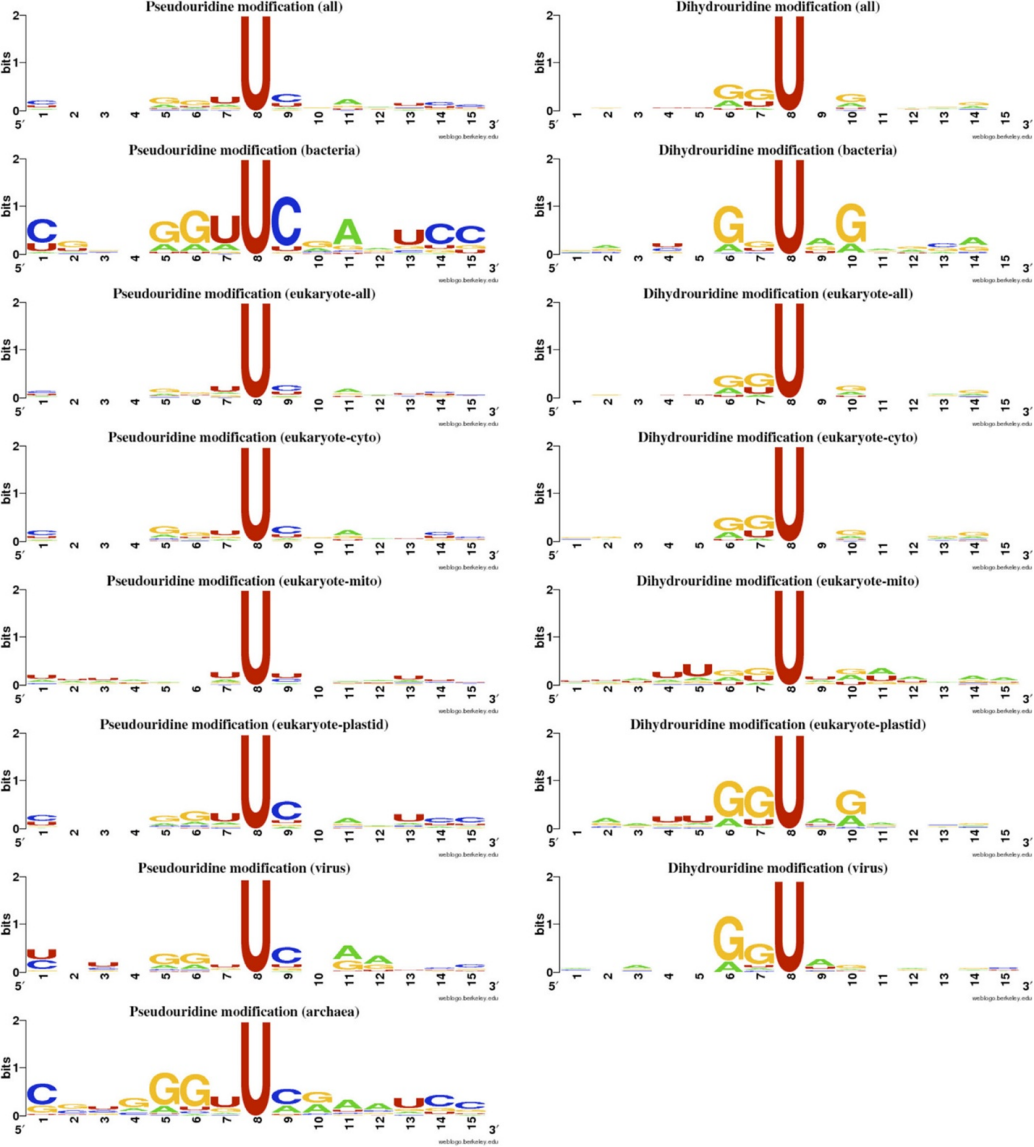
**Kingdom-wise WebLogos of pseudouridine and dihydrouridine using 15-length sliding window patterns (central 8th position for pseudouridine/dihydrouridine).**

There are several modifying enzymes that play important role in the post-transcriptional modifications of tRNA. It is important to investigate the differences between modified (29.27%) and unmodified uridines. Therefore, we created kingdom-wise TSLs and observed that significant differences were present between modified and unmodified uridines (Figure 2). The bases of central (8th) position were modified/unmodified uridines. The modified uridines were flanked by guanine (5′ end) and uridine (both 5′ and 3′ end) whereas unmodified uridines preferred cytosine (5′ end) and guanine (3′ end) as neighbors. Most of modified uridines preferred guanine at 6th and adenine at 11-12th positions (Figure 2). Although, there were 22 different type of uridine-modifications present but Pseudouridine (Y) was most (~45% of all UMs) abundant UMs (Additional file 1: Table S1). Sequence-based conservation of pseudouridine modifications was analyzed and observed that there was very low conservation present (Figure 3). Only some conservation of uridine at 5′ and cytosine at 3′end was present (especially in bacteria). It is well known because TYC (UUC in the WebLogo) is always present in TSL but pseudouridine is also present at other sites. The Dihydrouridine (D) was second most abundant (~32% of all UMs) and only present in the DSL but as WebLogos suggested that there was no sequence-based conservation present for this modification (Figure 3). On the basis of these analyses, we developed prediction models for the all uridine-modification, pseudouridine and dihydrouridine separately.

In this study, we used a three-step strategy to develop an efficient method for the prediction and classification of uridine modifications. In first step, we developed a common prediction method using tRNA-136 dataset of MODOMICS database update 2008 [39]. In the second step, we used newly added tRNA sequences (as independent dataset) of MODOMICS database update 2012 [40] for the kingdom-wise prediction performance evaluation of previously developed (in first step) common model. In the last step, we used tRNA-419 and tRNA-471 datasets (See details in the Methods section) of MODOMICS database update 2012 for the kingdom-wise prediction model development.

### Identification of uridine modifications

In past, various machine learning based prediction methods have been developed for biological problems and SVM is one of the most powerful and highly used algorithms. First, we generated sliding window of different lengths and created positive and negative patterns. If central nucleotide of window is modified uridine, the whole pattern was used as positive otherwise windows with unmodified uridines at central positions were used as negative patterns (see material and methods section). We optimized window sizes by prediction performances and applied following approaches and developed various SVM-based prediction models. In first step, all models were developed on a non-redundant dataset called ‘tRNA-136’ (see Methods section), which contain 136 tRNA sequences, where no two sequences have more than 50% sequence similarity.

#### Compositions-based approaches

We developed various SVM based modules for predicting modified uridine in tRNA using mono-, di- and tri-nucleotide composition (Additional file 1: Figures S3-S5) and optimized window size for achieving best performance in terms of area under curve (AUC). We achieved maximum AUC 0.76, 0.84 and 0.865 for mono-, di- and tri-nucleotide composition respectively.

#### Binary approach

The compositions-based approaches give information of only nucleotide frequencies; it has no information about sequential arrangement of these nucleotides. Therefore, we applied binary approach, which is widely used and is a successful strategy for the nucleotide (or residue) level predictions [42]. First, we generated binary profiles of patterns (BPP) of length 3 to 25 nucleotides. These BPPs were used to develop SVM based methods for predicting modified uridine in tRNA sequences. We computed performance of window length 3 to 25 and achieved maximum MCC 0.72 with accuracy 89.13% and AUC 0.924 at 17-window length (Additional file 1: Figure S6).

#### Structure-based approaches

All nucleotide sequences of tRNAs fold into well-defined cloverleaf like structures. There are loop-specific UMs presents in D-stem loop (DSL), T-stem loop (TSL), Anticodon-stem loop (ASL) and Variable loop (VL). Therefore, this structural information can also be useful for the prediction of UMs. We used three different software packages namely RNAfold [43], IPknot [44] and tRNAscan-SE [45] for predicting structure of tRNA. The binary representation of predicted tRNA structure was used for developing SVM based models (see material and methods section). The performance of SVM models were based on the binary representation of structures tRNA, predicted using RNAfold, IPknot and tRNAscan-SE shown in Additional file 1: Figures S7, S8 and S9 respectively. At window length 19, we achieved MCC 0.73 with AUC 0.925 for models based on predicted structures using tRNAscan-SE. One possible reason of better performance of tRNAscan-SE approach is that it was developed specifically for tRNA and it predicted DSL, ASL, VL and TSL boundaries correctly in comparison to RNAfold and IPknot.

#### Hybrid approach

**Figure 4:**
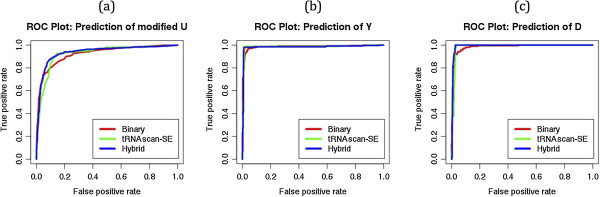
**ROC plots showing prediction performances of (a) all uridine, (b) pseudouridine and (c) dihydrouridine modifications using Binary, tRNAscan-SE and Hybrid approaches on the tRNA-136 dataset.**

As shown in above sections, SVM models based on BPP (window length 17) and on tRNAscan-SE (window length 19) predicted structures performed better than other models. In order to improve performance of our approach, we developed a model using windows-based five-fold cross validation that combines both types of information (see Methods section). This hybrid model performs better than existing models and achieved maximum 85.92% sensitivity, 91.68% specificity, 90.14% accuracy, MCC 0.76 and AUC of 0.936 (Figure 4a). We also used sequence-based five-fold cross validation of tRNA-136 dataset, where we kept all the windows of any single tRNA into a same sub-set during five-fold cross validation and achieved almost equal performance (83.13% Sensitivity, 92.36% Specificity, 89.84% accuracy and 0.75 MCC) to the window-based five-fold cross validation.

To check whether over-representation of Pseudouridine (Y), Dihydrouridine (D) and 5-methyl-uridine (T) caused any SVM paramer over-fitting or not, we have used almost equal representation of different modifications. We randomly selected 30 Y, 30 D, 30 T and 92 all other uridine modifications from the tRNA-136 dataset for model development. On this randomized dataset, we have achieved 85.72% sensitivity, 97.34% specificity, 96.27% accuracy, MCC 0.79 and AUC of 0.974 on the same previosuly optimized parameter (−z c -t 2 -g 0.05 -c 2 -j 2). It means there is no SVM parameter overfitiing during model development (Table 1).

**Table 1:** **SVM performance on the complete tRNA-136 and randomly selected tRNA-136 dataset using the same parameter (-z c -t 2 -g 0.05 -c 2 -j 2)**

Threshold	Complete tRNA-136 dataset				Randomized tRNA-136 dataset			
	SN	SP	ACC	MCC	SN	SP	ACC	MCC
**−1.00**	96.37	60.89	70.42	0.51	97.24	78.59	80.30	0.49
**−0.90**	95.31	70.16	76.91	0.58	96.68	85.80	86.80	0.58
**−0.80**	94.25	76.37	81.17	0.63	96.13	90.13	90.68	0.65
**−0.70**	93.64	80.42	83.97	0.67	***93.36***	***92.96***	***93.00***	***0.70***
**−0.60**	92.13	83.30	85.67	0.69	91.71	94.56	94.31	0.73
**−0.50**	91.37	85.14	86.81	0.71	89.52	95.73	95.16	0.76
**−0.40**	90.31	86.63	87.62	0.72	88.44	96.51	95.77	0.78
**−0.30**	89.40	87.80	88.23	0.73	87.34	96.89	96.02	0.78
**−0.20**	***88.64***	***89.35***	***89.17***	***0.75***	**85.72**	**97.34**	**96.27**	**0.79**
**−0.10**	87.43	90.57	89.73	0.75	84.07	97.67	96.42	0.79
**0.00**	**85.92**	**91.68**	**90.14**	**0.76**	80.80	98.17	96.58	0.79
**0.10**	83.80	92.57	90.22	0.75	75.82	98.56	96.47	0.78
**0.20**	81.08	93.01	89.81	0.74	74.71	98.78	96.58	0.78
**0.30**	79.72	93.62	89.90	0.74	71.44	98.89	96.37	0.77
**0.40**	76.70	94.34	89.61	0.73	68.69	99.00	96.22	0.76
**0.50**	73.67	95.28	89.49	0.72	62.69	99.11	95.77	0.72
**0.60**	69.43	95.89	88.80	0.70	58.27	99.33	95.57	0.70
**0.70**	65.20	96.67	88.23	0.69	53.87	99.33	95.16	0.67
**0.80**	59.00	97.28	87.01	0.65	45.11	99.39	94.41	0.61
**0.90**	*51.59*	*97.78*	*85.39*	*0.60*	32.40	99.45	93.30	0.50
**1.00**	40.84	98.39	82.95	0.53	26.37	99.72	93.00	0.46

Bold are performances with maximum MCC (Threshold 0.0 or close to 0.0 preferred).

Bold and italic are performances where gap between sensitivity and specificity are minimum.

### Classification of different uridine modifications

In the tRNA-136 dataset, ~72% of modified uridines belonged to either Pseudouridine or Dihydrouridine. Therefore, we developed separate prediction models for these two major classes of UMs.

#### Prediction of pseudouridine (Y) modification

We used patterns of Pseudouridine as positives and all other UMs as negatives. The maximum performance MCC 0.93 and 0.96 was achieved using SVM models based on BPP and tRNAscan-SE based approach respectively. We achieved best threshold-independent performance AUC 0.986, 0.985 and 0.983 using SVM models based on BPP, tRNAscan-SE predicted structures and hybrid approach respectively (Figure 4b).

#### Prediction of Dihydrouridine (D) modification

The patterns of Dihydrouridines were used as positive and other patterns of UMs were used as negatives. The BPP approach achieved 0.90 MCC and 0.986 AUC whereas tRNAscan-SE based approach achieved 0.95 MCC and 0.985 AUC. The hybrid approach predicted all Dihydrouridines correctly (100% sensitivity) with 97.36% specificity, 98.18% accuracy, 0.96 MCC and 0.991 AUC values (Figure 4c).

### Evaluation of developed models on the kingdom-wise independent datasets

**Figure 5:**
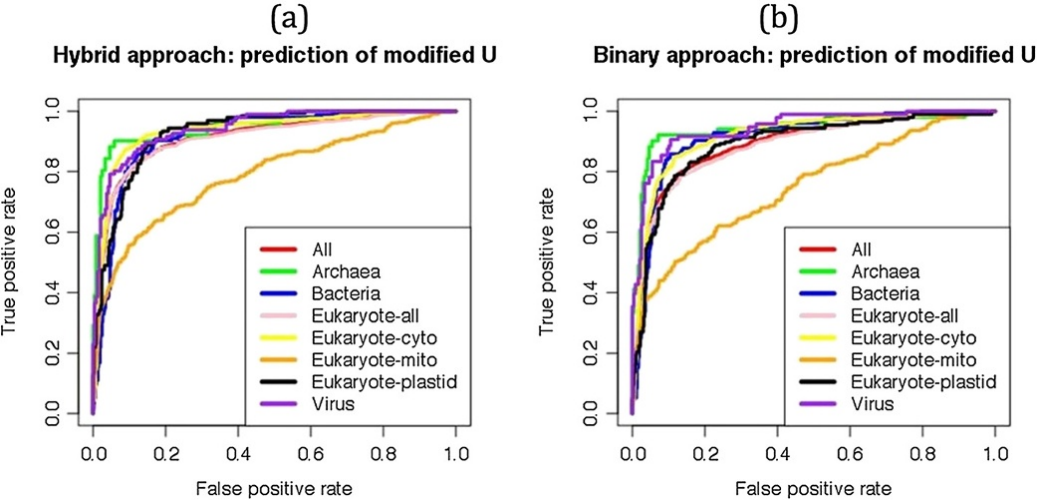
**ROC plots showing kingdom-wise prediction performances of (a) hybrid and (b) binary approach on the independent datasets.**

In the second step, we wanted to see the performance of a common prediction model (based on the tRNA-136 dataset) on the kingdom-wise independent datasets. We used newly added tRNA sequences (which were not used for the common prediction model development; see material and methods section) of MODOMICS databaset [40] update 2012, for the performance evaluation of previously developed model. We achieved 0.911, 0.949, 0.919, 0.910, 0.936, 0.789, 0.930 and 0.944 of AUC for the prediction of modified uridines of All, Archaea, Bacteria, Eukaryote-all, Eukaryote-cyto, Eukaryote-mito, Eukaryote-plastid and Viruses respectively (Figure 5a). The model was not good to predict UMs in the mitochondrial tRNAs because structure of 18 out of total 93 mitochondrial tRNAs were not predicted. Therefore, we also applied previously developed BPP approach based model (based on tRNA-136 dataset), where structural information was not required and achieved 0.892, 0.943, 0.916, 0.886, 0.919, 0.748, 0.892 and 0.945 of AUC for the prediction of modified uridines of All, Archaea, Bacteria, Eukaryote-all, Eukaryote-cyto, Eukaryote-mito, Eukaryote-plastid and Viruses respectively (Figure 5b). Here also prediction performance was low for mitochondrial tRNAs. In the most of cases performances were decreased when we applied BPP instead of hybrid approach. It means structural information provided important information for the both, common (all) and kingdom-wise predictions.

Hybrid approach based models achieved 0.84, 0.81, 0.90, 0.84, 0.82, 0.85, 0.97 and 0.75 of MCC for the pseudouridine prediction of All, Archaea, Bacteria, Eukaryote-all, Eukaryote-cyto, Eukaryote-mito, Eukaryote-plastid and Viruses respectively. In the dihydrouridine prediction, hybrid model achieved 0.90, 0.97, 0.89, 0.90, 0.88, 0.89 and 0.92 of MCC for the modified (dihydrouridine) uridines of All, Bacteria, Eukaryote-all, Eukaryote-cyto, Eukaryote-mito, Eukaryote-plastid and Viruses respectively.

### Kingdom-wise prediction model development

**Figure 6:**
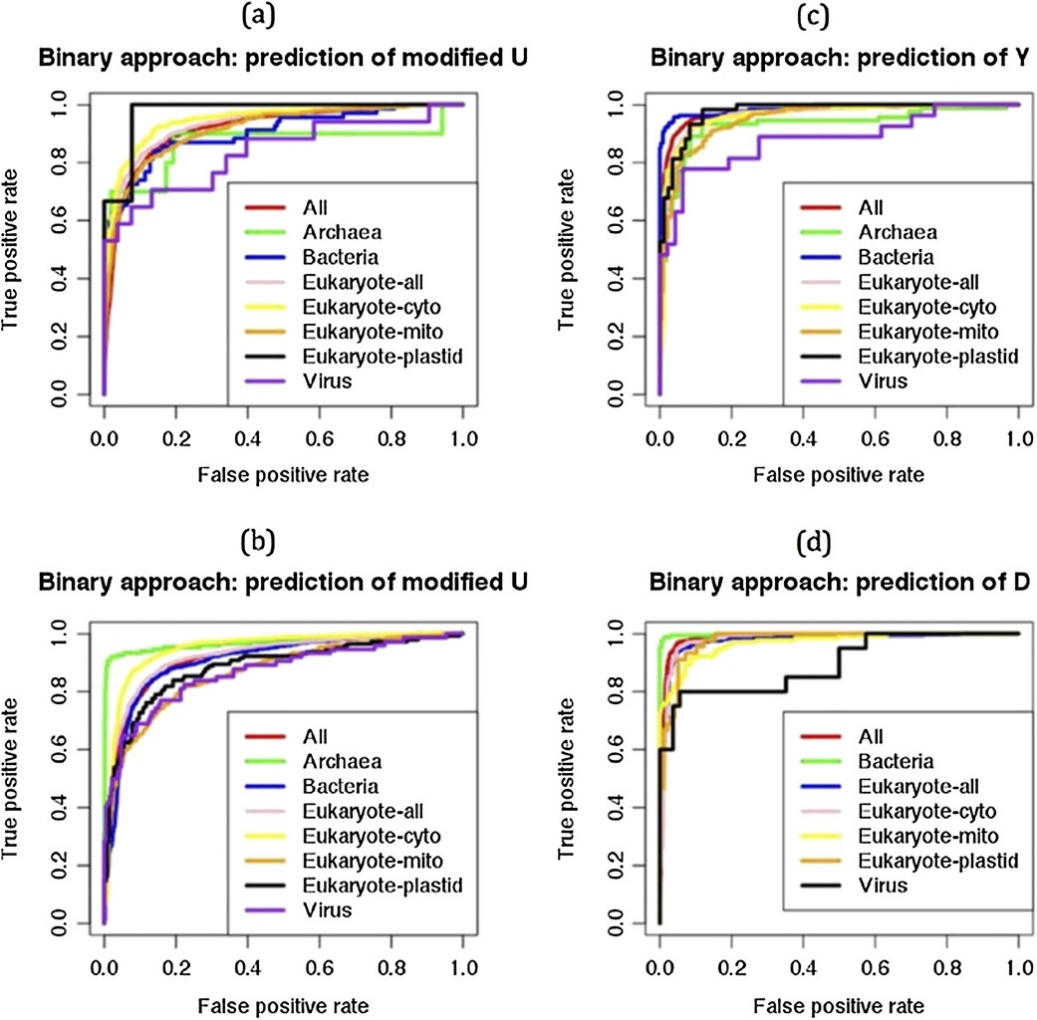
**ROC plots showing BPP approach based prediction performances on the kingdom-wise datasets (tRNA-471) for the prediction of (a) uridine modifications on the common prediction model, (b) uridine modifications on the kingdom-wise individual (separately for each kingdom/orgenelle) models, (c) Pseudouridine modification on the kingdom-wise separate models and (d) Dihydrouridine modification on the kingdom-wise separate models.**

In the third and last step, we used new kingdom-wise datasets from MODOMICS database [40] update 2012. It was shown in the previous approaches and evaluated by independent datasets that hybrid approach performed better if the structure predicted by tRNAscan-SE otherwise BPP approach can also be use as an alternative. Therefore, we have developed kingdom-specific prediction models using tRNA-419 and tRNA-471 datasets (See details in the material and method section). The tRNA-471 is a 50% non-redundant and containing 54 archaeal, 124 bacterial, 279 eukaryotic (142 cytosolic, 110 mitochondrial and 27 plastidic) and 14 viral tRNAs. First we developed a BPP approach based common prediction model for modified uridine prediction and achieved 0.917 of AUC. When we analyzed the kingdom-wise performance in this common prediction than we found 0.867, 0.901, 0.932, 0.946, 0.915, 0.974 and 0.837 of AUC for the Archaea, Bacteria, Eukaryote-all, Eukaryote-cyto, Eukaryote-mito, Eukaryote-plastid and Viruses respectively (Figure 6a). In the kingdom-wise individual (separately for each kingdom/orgenelle) model development, we achieved 0.970, 0.907, 0.925, 0.949, 0.868, 0.883 and 0.867 of AUC for the Archaea, Bacteria, Eukaryote-all, Eukaryote-cyto, Eukaryote-mito, Eukaryote-plastid and Viruses respectively (Figure 6b). These kingdom-wise individual models were also developed for the pseudouridine and dihydrouridine. In the pseudouridine prediction, we achieved 0.974, 0.933, 0.987, 0.964, 0.963, 0.952, 0.975 and 0.880 of AUC for the All, Archaea, Bacteria, Eukaryote-all, Eukaryote-cyto, Eukaryote-mito, Eukaryote-plastid and Viruses respectively (Figure 6c). We also achieved 0.987, 0.997, 0.977, 0.981, 0.970, 0.977 and 0.895 of AUC for the dihydrouridine prediction of All, Bacteria, Eukaryote-all, Eukaryote-cyto, Eukaryote-mito, Eukaryote-plastid and Viruses respectively (Figure 6d).

**Figure 7:**
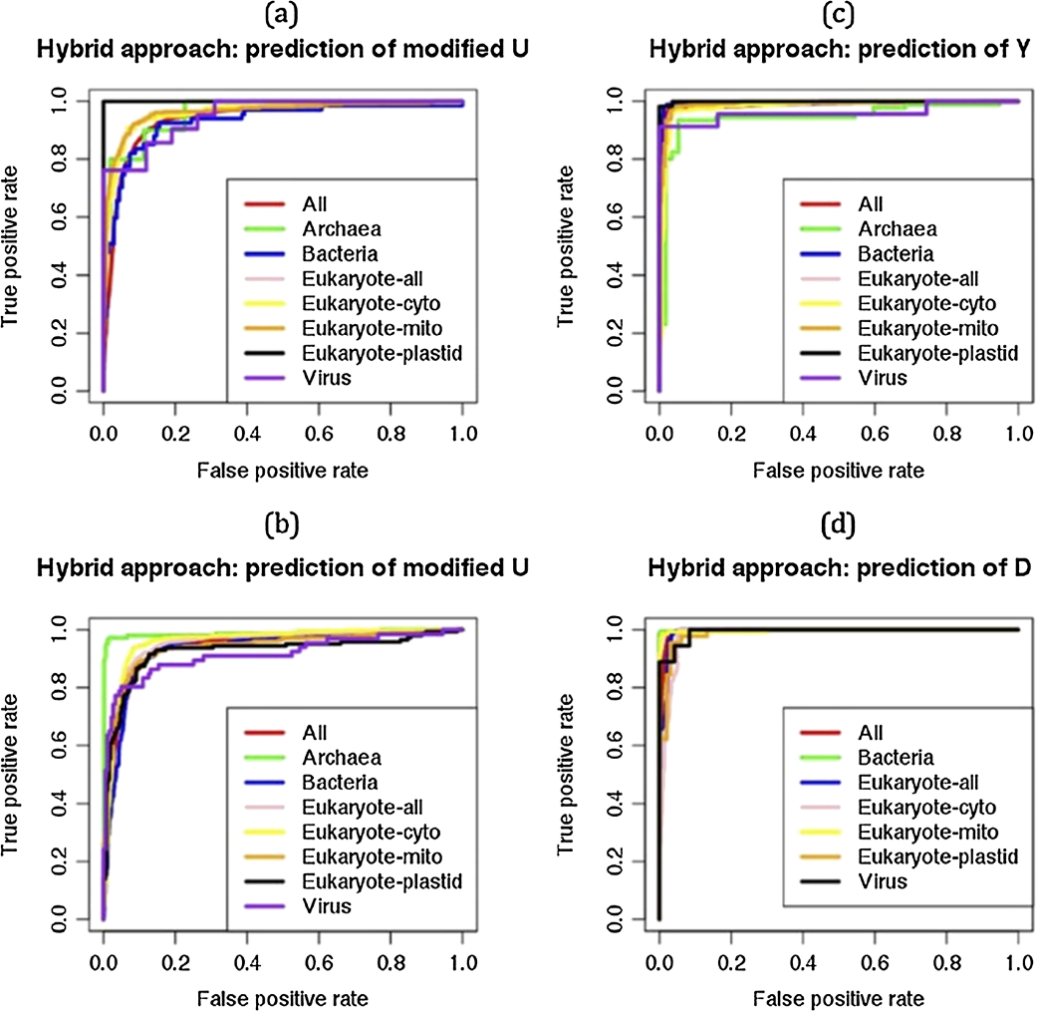
**ROC plots showing hybrid approach based prediction performances on the kingdom-wise datasets (tRNA-419) for the prediction of (a) uridine modifications on the common prediction model, (b) uridine modifications on the kingdom-wise individual (separately for each kingdom/orgenelle) models, (c) Pseudouridine modification on the kingdom-wise separate models and (d) Dihydrouridine modification on the kingdom-wise separate models.**

In the hybrid approach, we used tRNA-419 (50% non-redundant) dataset instead of tRNA-471, because tRNAscan-SE software was not predicted structures of 52 tRNA sequences. The tRNA-419 dataset containing 53 archaeal, 121 bacterial, 233 eukaryotic (114 cytosolic, 92 mitochondrial and 27 plastidic) and 12 viral tRNAs. A common prediction model for modified uridine prediction achieved 0.941 of AUC in comparison to 0.917 AUC of BPP approach. In this performance of 0.941 AUC, kingdom-wise prediction performances were 0.962, 0.930, 0.962, 0.962, 0.962, 1.00 and 0.952 of AUC for the Archaea, Bacteria, Eukaryote-all, Eukaryote-cyto, Eukaryote-mito, Eukaryote-plastid and Viruses respectively (Figure 7a). In the kingdom-wise individual model development, we achieved 0.987, 0.931, 0.953, 0.959, 0.940, 0.924 and 0.915 of AUC for the Archaea, Bacteria, Eukaryote-all, Eukaryote-cyto, Eukaryote-mito, Eukaryote-plastid and Viruses respectively (Figure 7b). These kingdom-wise individual models were also developed for the pseudouridine and dihydrouridine. The pseudouridine prediction performances were 0.986, 0.945, 0.996, 0.985, 0.981, 0.992, 0.999 and 0.961 of AUC for the All, Archaea, Bacteria, Eukaryote-all, Eukaryote-cyto, Eukaryote-mito, Eukaryote-plastid and Viruses respectively (Figure 7c). This hybrid approach achieved 0.994, 0.999, 0.990, 0.983, 0.997, 0.988 and 0.993 of AUC for the dihydrouridine prediction of All, Bacteria, Eukaryote-all, Eukaryote-cyto, Eukaryote-mito, Eukaryote-plastid and Viruses respectively (Figure 7d). The dihydrouridine modifications were absent in the archaeal tRNA sequences. These results showed that Hybrid approach performed better in comparison to BPP approach, whether kingdom-wise performances in the common method or individually developed methods. Hybrid approach also performed better than BPP for the prediction of pseudouridine and dihydrouridine.

Although, our main focused was to predict UMs in tRNA sequences and further classify them into pseudouridine and dihydrouridine modifications but we also tried to develop method for the third (~11% of all UMs) most abundant UM- 5-methyl-uridine.

### Prediction of 5-methyl-uridine (T) modification

The tRNA-136 dataset contains 14.2% UMs as 5-methyl-uridines. We analyzed kingdom-wise patterns and found that this modification is present only at a well-known conserved site GTYC (GUUC in the WebLogo) of the T-Stem Loop (Additional file 1: Figure S10). Therefore, it is easy to predict this modification, if boundaries of loops (mainly VL and TSL) are correctly predicted in the tRNA. The patterns of 5-methyl-uridine were used as positives and other UMs were used as negatives for developing SVM-based models. First we have used tRNA-136 and developed common model for the prediction of 5-methyl-uridines. In the threshold-dependent performances, BPP approach achieved 97.9% sensitivity, 97.36% specificity, 97.43% accuracy and 0.90 MCC whereas tRNAscan-SE achieved 96.84% sensitivity, 94.54% specificity, 94.86% accuracy and 0.82 MCC. Hybrid approach increased the performance and achieved 94.74% sensitivity, 98.42% specificity, 97.88% accuracy and 0.92 MCC. The threshold-independent performance of BPP, tRNAscan-SE and Hybrid approaches achieved 0.991, 0.971 and 0.993 AUC values (Additional file 1: Figure S11a). On the independent datasets (which were not used in the tRNA-136 dataset), Hybrid approach based model achieved 0.90, 0.97, 0.93, 0.93, 0.98, 0.90 and 0.78 of MCC for the 5-methyl-uridine prediction of All, Bacteria, Eukaryote-all, Eukaryote-cyto, Eukaryote-mito, Eukaryote-plastid and Viruses respectively. Finally, we applied BPP (tRNA-471 dataset) and hybrid (tRNA-419 dataset) approaches and achieved 0.993, 0.997, 0.996, 0.997, 0.997, 1.00 and 0.985 of AUC for BPP (Additional file 1: Figure 11b) and 0.993, 0.995, 0.996, 0.996, 0.998, 1.00 and 0.985 of AUC for hybrid approach (Additional file 1: Figure 11c) for the All, Bacteria, Eukaryote-all, Eukaryote-cyto, Eukaryote-mito, Eukaryote-plastid and Viruses respectively. The 5-methyl-uridine modification was absent in the archaeal tRNA sequences.

### Prediction of other uridine modifications

To see whether SVM based machine learning can discriminate these three (Y, D and T) modifications from other UMs (remaining ~12%) or not, we applied same above-mentioned strategy. We used tRNA-136 dataset and patterns of Y, D and T as negatives and patterns of other UMs as positives. The BPP approach performed 66.26% sensitivity, 98.42% specificity, 93.95% accuracy and 0.73 MCC. The structural information of tRNAscan-SE based approach achieved 52.16% sensitivity, 99.82% specificity, 93.19% accuracy and 0.69 MCC. The Hybrid approach increased performance significantly and achieved 75.96% sensitivity, 97.89% specificity, 94.86% accuracy and 0.78 MCC. The threshold-independent performance of BPP, tRNAscan-SE and Hybrid approaches achieved 0.924, 0.868 and 0.922 AUC values (Additional file 1: Figure S12a).

On the independent datasets, Hybrid approach based model achieved 0.58, 0.77, 0.86, 0.41, 0.38, 0.49, 0.53 and 0.64 of MCC for the other modified (except Y, D and T) uridines of All, Archaea, Bacteria, Eukaryote-all, Eukaryote-cyto, Eukaryote-mito, Eukaryote-plastid and Viruses respectively. The BPP (tRNA-471 dataset) and hybrid (tRNA-419 dataset) approaches were achieved 0.905, 0.933, 0.974, 0.845, 0.847, 0.916, 0.905 and 0.860 of AUC for BPP (Additional file 1: Figure 12b) and 0.925, 0.947, 0.976, 0.889, 0.877, 0.967, 0.867 and 0.995 of AUC for hybrid approach (Additional file 1: Figure 12c) for the All, Archaea, Bacteria, Eukaryote-all, Eukaryote-cyto, Eukaryote-mito, Eukaryote-plastid and Viruses respectively. The prediction performances were low in comparison to Y, D and T because here we used total 19 different types of modifications together as positives. In order to implement the prediction model in the form of web-server, it was necessary to develop a separate prediction model for other UMs (remaining ~12%).

In conclusion, SVM-based prediction modules performed better with hybrid approach of BPP and tRNAscan-SE based structural information. The details of all results given in an excel file (see Additional file 2), contain results of all approaches, window sizes at all (−1.0 to 1.0) thresholds and ROC graphs.

## Discussion

**Figure 8:**
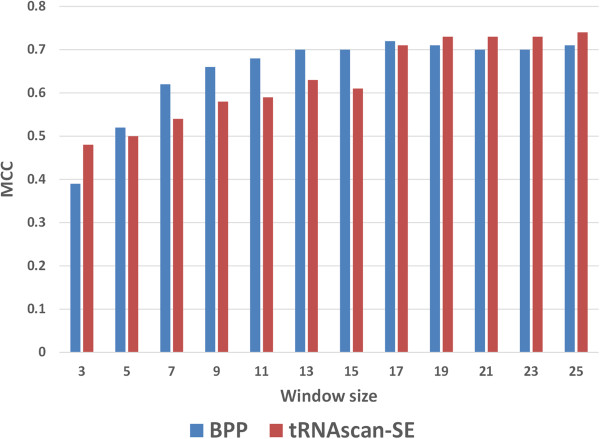
**Prediction performance (MCC) of BPP and tRNAscan-SE based approaches using different window sizes.**

In present study, we retrieved information of 218 and 642 modified tRNAs from MODOMICS database [39, 40]. Initially, we analyzed all 642 tRNA modifications and observed that majority (55.85%) of modifications were uridine-derived (Figure 1) and 29.27% uridines of all uridines were modified. Therefore, we selected only UMs for further study. The kingdom-wise differences between flanking nucleotides of modified and unmodified uridines were observed (Figure 2). It may be due to the pattern-wise preference of modifying enzymes. In past, sliding window-based approach was widely used for nucleotide/residue level predictions [46]. It requires complete optimization of all window sizes for every prediction. We created different lengths (3–25) of sliding window patterns and various approaches of compositions; BPP and structural information were applied. First we used tRNA-136 dataset for the common prediction model and evaluated using 5-fold cross validation technique. In compositions based input features of MNC, DNC and TNC achieved AUC of 0.76, 0.840 and 0.865 respectively. The BPP increased the prediction performance to AUC of 0.924 AUC because it provided information of nucleotides with their positions whereas compositions based approaches have only frequencies or one/two neighboring nucleotide information. All tRNA fold into well defined structures with some regions and loops more prone to modification thereby making the structural information useful for prediction. Consequently, secondary structures provided by tRNAscan-SE software achieved AUC of 0.925. When the structural information of tRNAscan-SE software predicted boundaries of different loops was combined with positional information of nucleotides in the form of BPP; performance increased significantly and achieved AUC of 0.936 (Figure 4a). We have also analyed the effect of window size on the prediciton performance and found that performance continously increased in 3–15 window size and saturated between the 17–25 window sizes (Figure 8).

In the second step, we evaluated previously developed (based on tRNA-136) models on the independent datasets and analyzed the kingdom wise performances. Hybrid approach performed well for All, Archaea, Bacteria, Eukaryote-all, Eukaryote-cyto, Eukaryote-plastid and Viruses but not performed for Eukaryote-mito (Figure 5). It may be because tRNA-136 contained only 18 mitochondrial tRNAs of *Saccharomyces cerevisiae* and developed model was not good enough to predict UMs in the mitochondrial tRNAs from other eukaryotes. Although, mitochondrial tRNAs have evolutionary connection with bacterial tRNAs but our results showed that prokaryotic datasets based model cannot predict the uridine modifications in the mitochondrial tRNAs.

In the last step, we used BPP (based on tRNA-471 dataset) and hybrid (based on tRNA-419 dataset) approach for the final kingdom-specific prediction models and evaluated using five-fold cross validation technique. The hybrid approach performed better than simple BPP approach in all kingdoms. It means structural information provided important information, when it integrated with BPP (Figures 6 and 7). Hybrid approach achieved 0.941 AUC for all tRNAs but when applied separately for Archaea and Eukaryotes, it increased to the AUC of 0.987 and 0.953 respectively. We found that hybrid approach of BPP and tRNAscan-SE was efficient not only to predict UMs but also to classify them into pseudouridine and dihydrouridine (Figure 7). The BPP approach can also be useful as an alternative, if structure of tRNA is not available (Figure 6). We also developed models for the third most abundant (~11% of all UMs) 5-methyl-uridines UMs.

A recent study of Novoa et al. [38] showed that additional information of two modifications (I_34_ and xo^5^U_34_) improved correlation between codon usage and tRNA gene frequencies in all kingdoms significantly. Modifications in ASLs are very important given the fact that modified U_34_ acts as proton donor/acceptor, coordinates metal ions and has great diverse chemistry [27] ultimately affecting codon-anticodon recognition. The ASLs, DSLs, TSLs and VLs of different tRNAs vary in sequence and the type of modification. Modification of seven-nucleotide ASLs (especially wobble 34 and purine 37 site) is more important because global conformation of ASLs decides entry of anticodon domain into the ribosomal A-site [13] and affects translation speed [47]. Data availability is the main criterion for the development of any prediction model and the number of uridine modifications (55.85%) is very high in comparison to guanine (19.71%), adenine (12.28%) and cytosine (10.41%) modifications. Therefore, we developed prediction models for UMs only. Many RNA modifications are not essential for cell survival. Probably these modifications are less important or not fully explored yet considering the fact that many DNA and protein modifications are also not essential. Precise roles of most of tRNA modifications are poorly understood and their industrial applications are still unexplored. Pseudouridine provides rigidity whereas dihydrouridine is the only non-aromatic nucleoside and provides flexibility to the tRNA structure. In this era of synthetic biology, better understanding of tRNA modifications will help in the better tRNA designing, incorporation of novel amino acids and production of new proteins. In particular, biochemists have great opportunity to play with the chemistry of wobble base and expand amino acid boundaries.

## Conclusion

To conclude, the present study is a systematic attempt to predict and classify UMs in tRNA sequences. We developed separate and kingdom-wise predictors for the prediction of UMs and thereafter classify them into Pseudouridines, Dihydrouridines and 5-methyl-uridine and other UMs. We found that hybrid approach of binary and structural information is most suitable for the SVM based prediction of UMs. These prediction modules have been implemented in a ‘tRNAmod’ web-server. This server can predict and classify UMs from tRNA sequences or whole genome of any organism.

## Methods

### Datasets

In this study, two different version of MODOMICS database update 2008 [39] and update 2012 [40] have been used. Update 2008 and 2012 of MODOMICS database were containing 218 and 642 tRNA (modified) sequences respectively.

#### MODOMICS database update 2008

We extracted 43 different types of modification in total 218 (35 *Bacillus subtilis*, 47 *Escherichia coli*, 41 *Halobacterium volcanii*, 29 *Mycoplasma capricolum* and 66 *Saccharomyces cerevisiae*) modified tRNA sequences from MODOMICS database [39]. Out of the total 17088 bases in these sequences, 1674 bases are modified and 15414 are unmodified. The base-specific contribution in modifications of uridine, guanine, adenine and cytosine are 61.4%, 17.2%, 11.2% and 10.2% respectively.

#### tRNA-136

To develop a prediction method, it is important to create non-redundant dataset because prediction of modified base in diverse sequences is a major challenge. In the case of RNA sequences 50% non-redundancy level is sufficient to evaluate prediction model and also most of tools are not reliable for generating more stringent redundancy level for nucleotide sequences. Therefore, we created 50% non-redundant (NR) dataset of 136 (16 *Bacillus subtilis*, 33 *Escherichia coli*, 31 *Halobacterium volcanii*, 9 *Mycoplasma capricolum* and 47 *Saccharomyces cerevisiae*) tRNA sequences from MODOMICS database update 2008 using BLASTCLUST software and termed it as ‘tRNA-136’. The tRNA-136 dataset contains a total of 10654 bases, out of which 1095 are modified (10.28%) and 9559 are unmodified bases. It includes 40 different types of modifications. In tRNA-136 dataset, 661 (60.4%) modified-bases are uridine-derived. Most of uridine modifications belong to Pseudouridine (40.5%), Dihydrouridine (31.3%), 5-methyl-uridine (14.2%) and remaining 14% are other UMs.

#### MODOMICS database update 2012

We extracted total 642 (413 eukaryotic, 152 bacterial, 60 archaeal and 17 viral) modified tRNA sequences from MODOMICS database [40], which contained ~60 type of modifications from 77 different organisms (49 eukaryotes, 17 bacteria, 7 archaea and 4 viruses). Total ~13% bases were modified and uridine-, guanine-, adenine- and cytosine-derived modified bases were 55.85%, 19.71%, 12.28% and 10.41% of total modified bases respectively (Additional file 1: Table S1).

#### Independent datasets

In order to create kingdom-wise independent datasets, we used only newly updated (organism-wise) tRNA sequences of MODOMICS database update 2012 and excluded all the tRNA sequences (all the tRNAs of *Bacillus subtilis*, *Escherichia coli*, *Haloferax volcanii* (formerly named as *Halobacterium volcanii)*, *Mycoplasma capricolum* and *Saccharomyces cerevisiae*) of MODOMICS update 2008. In this way, we created kingdom-wise independent datasets of all 407 tRNAs, which contains 19 archaeal, 41 bacterial, 330 eukaryotic (199 cytosolic, 93 mitochondrial and 38 plastidic) and 17 viral tRNA sequences. These independent datasets were used for the kingdom-wise performance evaluation of common prediction model (based on tRNA-136).

#### tRNA-471

We used all 642 tRNAs of MODOMICS database update 2012 and created 50% non-redundant (NR) dataset of 471 tRNA sequences using BLASTCLUST software and termed it as ‘tRNA-471’. It contains 54 archaeal, 124 bacterial, 279 eukaryotic (142 cytosolic, 110 mitochondrial and 27 plastidic) and 14 viral tRNA sequences. We used these kingdom-wise datasets for the model development of BPP approach.

#### tRNA-419

In the hybrid approach, tRNAscan-SE software was not predicted structure of 52 tRNAs from tRNA-471 dataset. Therefore, we created separate dataset of 419 tRNAs and termed as ‘tRNA-419’. It contains 53 archaeal, 121 bacterial, 233 eukaryotic (114 cytosolic, 92 mitochondrial and 27 plastidic) and 12 viral tRNA sequences. In the hybrid approach, we used these kingdom-wise datasets for the prediction model development.

Both the dataset, data-218 (MODOMICS database update 2008) and data-642 (MODOMICS database update 2012) have been provided in the Additional file 3 and Additional file 4 respectively. The nomenclature of modification used from MODOMICS database, which has been also provided in the Additional file 1: Table S1.

### Creation of sliding windows

In past, sliding window-based strategies have been successfully applied for residue level predictions [46]. Thus, we created and optimized different lengths of window size (3–25) of odd numbers (eg. 3, 5, 7,…, 25). If the central nucleotide of window pattern is modified then it was assigned as positive pattern otherwise assigned as negatives. In this way, we created window patterns for each nucleotide in the tRNA-136 dataset. To generate fixed length window size of terminal nucleotides, we added a dummy ‘X’ nucleotide in both terminals of each sequence. The number of dummy nucleotides was calculated with (L-1)/2 formula (where L is the length of pattern). In this study, we used maximum window size 25 because the average length of tRNA is 77 (each containing 3 loops) and large window sizes are not advisable for better machine learning. Thus, we preferred well performing small window to large window sizes.

### Compositions-based approaches

In this approach, we calculated mono-nucleotide composition (MNC), di-nucleotide composition (DNC) and tri-nucleotide composition (TNC) of tRNA sequences. After adding dummy ‘X’ at both terminals, total number of nucleotides reached to five (A, C, G, U and X). The MNC, DNC and TNC generated input features of 5 (A, C, G, U and X), 25 (AA, AC, AG, CG, AU,…, XX) and 125 (AAA, AAC, AAG,…, XXX) dimensions of vector.

### Binary approach

As discussed above, positive and negative patterns of sliding windows were created but numerical representation of these patterns is necessary for SVM-based machine learning. Thus, we applied binary profile of patterns (BPP) approach, which gives nucleotide (or amino acids) information as well their positional (sequential) information [46]. In BPP, we represented A, C, G, U and X nucleotides with {1,0,0,0,0}, {0,1,0,0,0}, {0,0,1,0,0}, {0,0,0,1,0} and {0,0,0,0,1} respectively. The number of total input features generated by BPP was five times more than used window size (e.g. 17-length window generates total 85 (17×5) input features).

### Structure-based approaches

All tRNA sequences fold into well-defined structures, thus structural information was used for SVM-based machine learning. The secondary structures of all tRNAs were predicted using three different software: RNAfold [43], IPknot [44] and tRNAscan-SE [45]. We converted all the secondary structural information into binary pattern for prediction model development. Here also dummy ‘X’ were used for the terminal nucleotides in order to create fixed length patterns. RNAfold gives three types of secondary structures, thus we represented small open bracket, small close bracket, dot and X as {1,0,0,0}, {0,1,0,0}, {0,0,1,0} and {0,0,0,1} respectively. IPknot predicted five different types secondary structures, which include additional information of pseudoknots in the form of square brackets (open and close). Thus, binary representations of small open bracket, small close bracket, square open bracket, square close bracket, dot and dummy X were {1,0,0,0,0}, {0,1,0,0,0}, {0,0,1,0,0}, {0,0,0,1,0} and {0,0,0,0,1} respectively. The tRNAscan-SE predicts tRNA secondary structures specifically and gives three different types secondary structures. We represented <, >, dot and X in the binary form of {1,0,0,0}, {0,1,0,0}, {0,0,1,0} and {0,0,0,1} respectively. In this way, we converted all the secondary structural information into machine learning input format and developed separate prediction model for each software.

### Hybrid approach

The hybrid approach is an integration of two or more approaches. In this study, we observed that binary information of BPP (85 features of 17-length window) and predicted structures of tRNAscan-SE (76 input features of 19-length window) performed well individually. Thus, we integrated these two approaches and created 161 (85 + 76) input features. This hybrid approach, provided both nucleotide-wise positional information and structural information further improving prediction performance.

### Support vector machine

SVM is widely used and highly successful machine learning technique for the biological predictions [48, 49]. It is based on the structural risk minimization principle of statistical learning theory. SVMs are a set of supervised learning methods, which can be used for both classification and regression mode [50]. We implemented SVM^light^ (version 6.02) package for the development of all prediction models [51]. It provides several parameters and kernels (e.g. linear, polynomial, radial basis function, and sigmoid) or any user-defined kernel. The svm_learn and svm_classify are two main softwares in this package. First, we used svm_learn for training of known examples and building of prediction models. After training, learned models predicted unknown examples (in five-fold cross-validation) using svm_classify. We tried various parameters and kernels and found that radial basis function (RBF) kernel performed well in all cases.

### Five-fold cross validation

In past, various prediction performance evaluation techniques have been applied such as leave-one out cross-validation (LOOCV) or jack-knife test, n-fold cross validation technique [52]. Though, jack-knife test is best for performance evaluation but it is a time-consuming process. Thus, we used 5-fold cross validation technique, which is highly used in the performance evaluation of biological predictions [53, 54]. In 5-fold cross validation, we divided both positive and negative samples into five subsets separately. We created five sets and each set containing one positive and one negative subset. The four subsets have been used for training and the remaining fifth subset was used for testing and calculating the performance. This step was repeated five times in such a way that each subset was used once for testing. The final performance is an average performance of all five testing sets.

### Evaluation parameters

The prediction performance of each model was calculated in the form of sensitivity (Equation 1), specificity (Equation 2), accuracy (Equation 3) and MCC (Equation 4) values. These are well-established evaluation parameters for biological prediction [54].
1234

Where TP, TN, FP and FN are True Positives, True Negative, False Positives and False Negatives respectively.

Above-mentioned evaluation parameters are threshold-dependent so we also calculated threshold-independent performance in terms of Area Under Curve (AUC) values using Receiver Operating Curve (ROC) plots. It is a plot between true positive rate and false positive rate.

The tRNAmod web-server gives probability score for each prediction. To calculate this score we used Equation 5, where SVM score of more than 1.5 and less than −1.5 was fixed to 1.5 and −1.5 respectively.
5

The probability score range varies from 0–9 only. We adopted this strategy because it is easy to display this probability score with tRNA sequence in tRNAmod web-server.

### Description of tRNAmod web-server

A user-friendly web-server tRNAmod was developed for the kingdom-wise prediction of UMs in tRNA sequence. There are two different prediction options available in tRNAmod, (1) Sequence-level prediction and (2) Genome-wide prediction. In the sequence-level prediction, it requires tRNA sequences in FASTA format and it will directly predict UMs in the given tRNAs. In the Genome-wide prediction, it requires whole genome sequence (also in FASTA fromat). First, it will extract tRNA sequences from the submitted genome using tRNAscan-SE [45] software then tRNAmod will predict UMs in extracted tRNA sequences. It will show the probability score (ranges 0–9) for each predicted UMs. The server shows secondary structure information of tRNA using tRNAscan-SE. A java-based applet VARNA [55] also has been implemented for structure visualization of tRNA, where predicted UMs will be highlighted in the tRNA structure. The tRNAmod is freely available for the help of global scientific community and is available at http://crdd.osdd.net/raghava/trnamod.

## Electronic supplementary material

Additional file 1: Figure S1: Weblogo of tRNA sequences in the standard representation of 1–99 positions. **Figure S2.** Kingdom-wise percent distribution of different base-specific modifications. **Figure S3.** A ROC plot showing prediction performances of uridine modifications of different window sizes using MNC approach. **Figure S4.** A ROC plot showing prediction performances of uridine modifications of different window sizes using DNC approach. **Figure S5.** A ROC plot showing prediction performances of uridine modifications of different window sizes using TNC approach. **Figure S6.** A ROC plot showing prediction performances of uridine modifications of different window sizes using binary approach. **Figure S7.** A ROC plot showing prediction performances of uridine modifications of different window sizes using RNAfold based approach. **Figure S8.** A ROC plot showing prediction performances of uridine modifications of different window sizes using IPknot based approach. **Figure S9.** A ROC plot showing prediction performances of uridine modifications of different window sizes using tRNAscan-SE based approach. **Figure S10.** Kingdom-wise WebLogos of 5-methyl-uridine using 15-length sliding window patterns (central 8th position for 5-methyl-uridine). **Figure S11.** ROC plots showing performances for the prediction of 5-methyl-uridine (T) on the (a) tRNA-136 dataset, (b) BPP appraoch of tRNA-471 dataset and (c) hybrid approach of tRNA-419 dataset. **Figure S12.** ROC plots showing performances for the prediction of other uridines (except Y, D and T) modifications on the (a) tRNA-136 dataset, (b) BPP appraoch of tRNA-471 dataset and (c) hybrid approach of tRNA-419 dataset. **Table S1.** Modification-wise distribution of 642 tRNAs of the MODOMICS database. The nomenclature of modification used from MODOMICS database. (DOC 3 MB)

Additional file 2: **Contains the detailed results of different approaches at different window sizes and thresholds.** (XLS 891 KB)

Additional file 3: **Contains the information of 218 modified tRNA from MODOMICS database update 2008.** (TXT 45 KB)

Additional file 4: **Contains the information of 642 modified tRNA from MODOMICS database update 2012.** (TXT 133 KB)
