# Supplementary material for: Prediction of uridine modifications in tRNA sequences
Source: BMC Bioinformatics. 2014 Oct 2;15(1):326. doi: 10.1186/1471-2105-15-326 (PMC4287530; doi:10.1186/1471-2105-15-326)
Supplement: Supplementary file 1 — Additional file 1: Figure S1: Weblogo of tRNA sequences in the standard representation of 1–99 positions. Figure S2. Kingdom-wise percent distribution of different base-specific modifications. Figure S3. A ROC plot showing prediction performances of uridine modifications of different window sizes using MNC approach. Figure S4. A ROC plot showing prediction performances of uridine modifications of different window sizes using DNC approach. Figure S5. A ROC plot showing prediction performances of uridine modifications of different window sizes using TNC approach. Figure S6. A ROC plot showing prediction performances of uridine modifications of different window sizes using binary approach. Figure S7. A ROC plot showing prediction performances of uridine modifications of different window sizes using RNAfold based approach. Figure S8. A ROC plot showing prediction performances of uridine modifications of different window sizes using IPknot based approach. Figure S9. A ROC plot showing prediction performances of uridine modifications of different window sizes using tRNAscan-SE based approach. Figure S10. Kingdom-wise WebLogos of 5-methyl-uridine using 15-length sliding window patterns (central 8th position for 5-methyl-uridine). Figure S11. ROC plots showing performances for the prediction of 5-methyl-uridine (T) on the (a) tRNA-136 dataset, (b) BPP appraoch of tRNA-471 dataset and (c) hybrid approach of tRNA-419 dataset. Figure S12. ROC plots showing performances for the prediction of other uridines (except Y, D and T) modifications on the (a) tRNA-136 dataset, (b) BPP appraoch of tRNA-471 dataset and (c) hybrid approach of tRNA-419 dataset. Table S1. Modification-wise distribution of 642 tRNAs of the MODOMICS database. The nomenclature of modification used from MODOMICS database. (DOC 3 MB) [file 12859_2014_6647_MOESM1_ESM.doc]

**SUPPLEMENTARY MATERIALS**

**Title of manuscript:** Prediction of uridine modifications in tRNA sequences

**Name of authors:** Bharat Panwar and Gajendra P. S. Raghava

**Name of file:** Supplementary_file1.doc

**(A) Supplementary Figures**

**
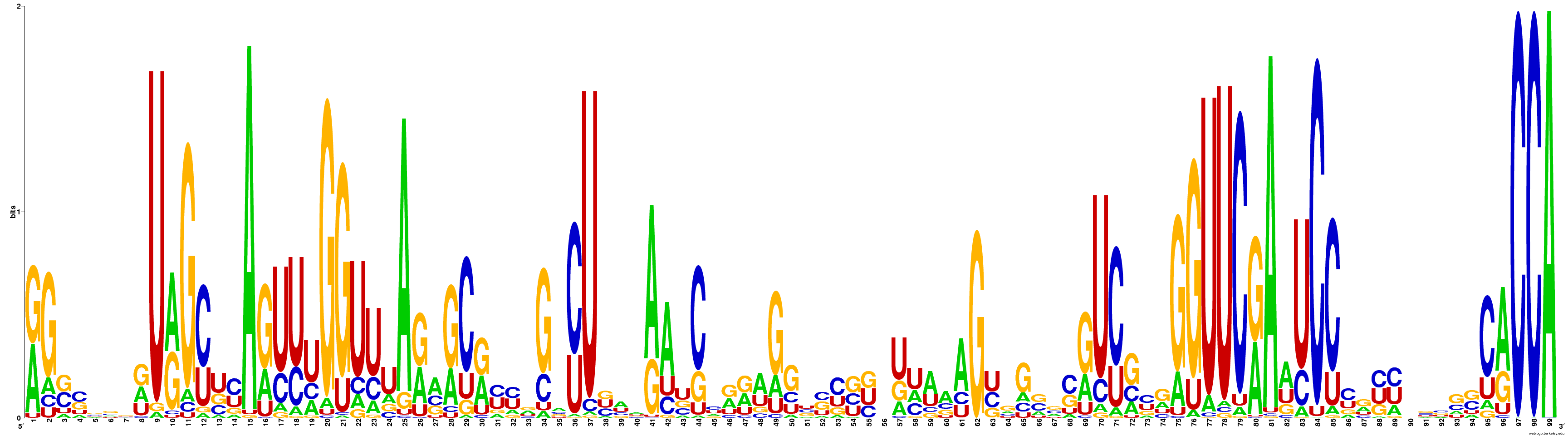
**

**Figure S1: Weblogo of tRNA sequences in the standard representation of 1-99 positions.**

**
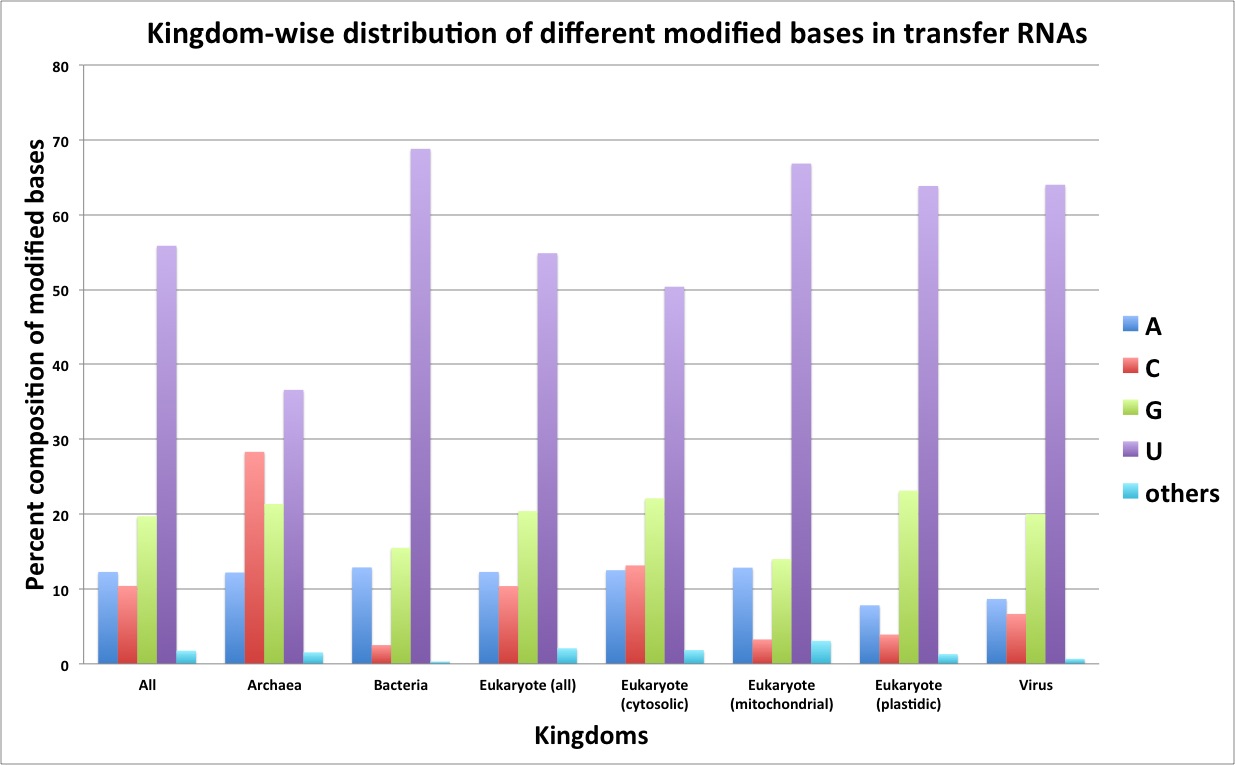
**

**Figure S2: Kingdom-wise percent distribution of different base-specific modifications.**

**
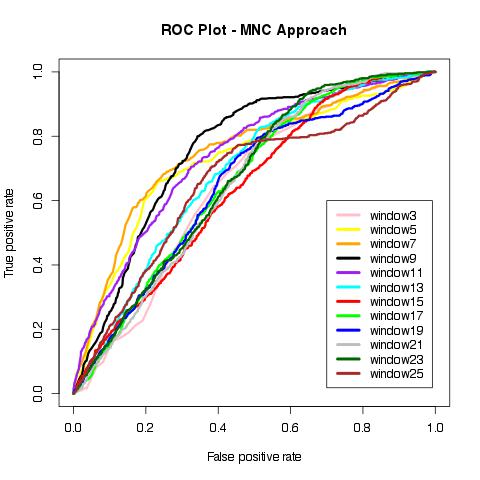
**

**Figure S3: A ROC plot showing prediction performances of uridine modifications of different window sizes using MNC approach.**

**
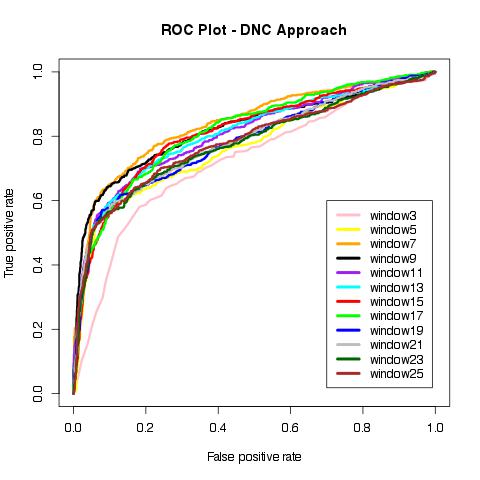
**

**Figure S4: A ROC plot showing prediction performances of uridine modifications of different window sizes using DNC approach.**

**
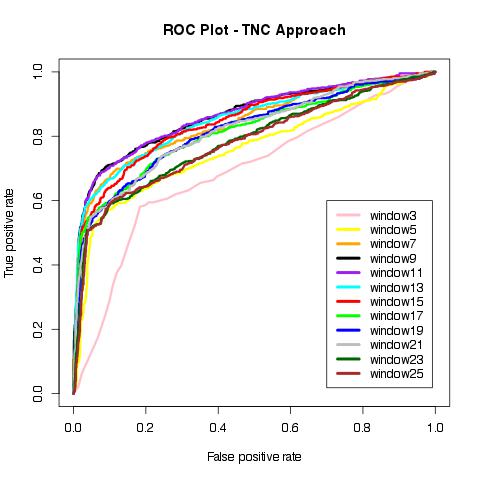
**

**Figure S5: A ROC plot showing prediction performances of uridine modifications of different window sizes using TNC approach.**

**
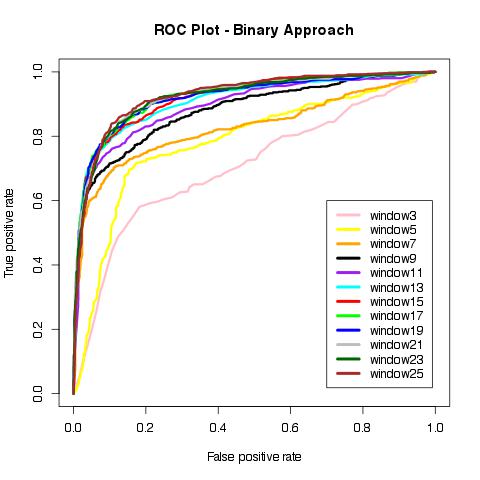
**

**Figure S6: A ROC plot showing prediction performances of uridine modifications of different window sizes using binary approach.**

**
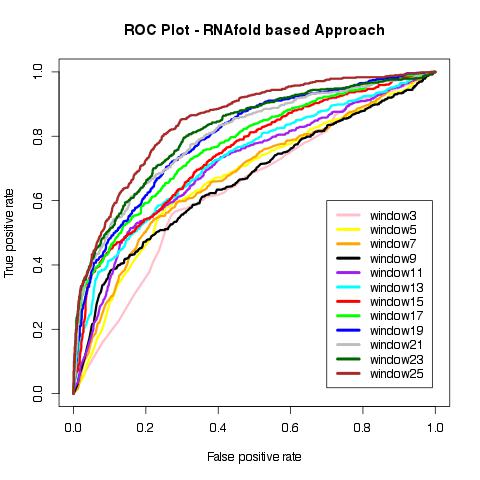
**

**Figure S7: A ROC plot showing prediction performances of uridine modifications of different window sizes using RNAfold based approach.**

**
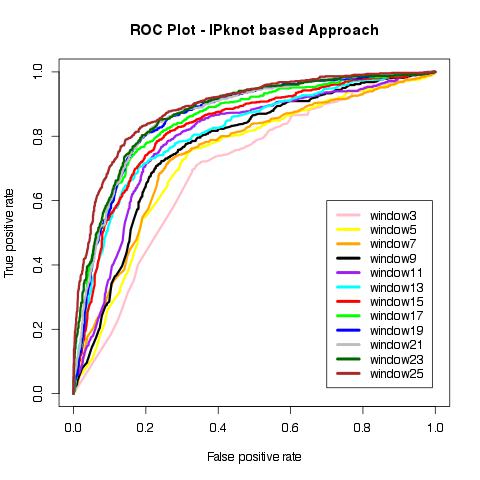
**

**Figure S8: A ROC plot showing prediction performances of uridine modifications of different window sizes using IPknot based approach.**

**
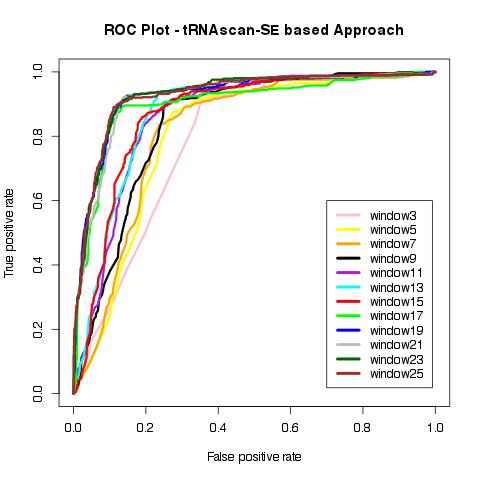
**

**Figure S9: A ROC plot showing prediction performances of uridine modifications of different window sizes using tRNAscan-SE based approach.**

**
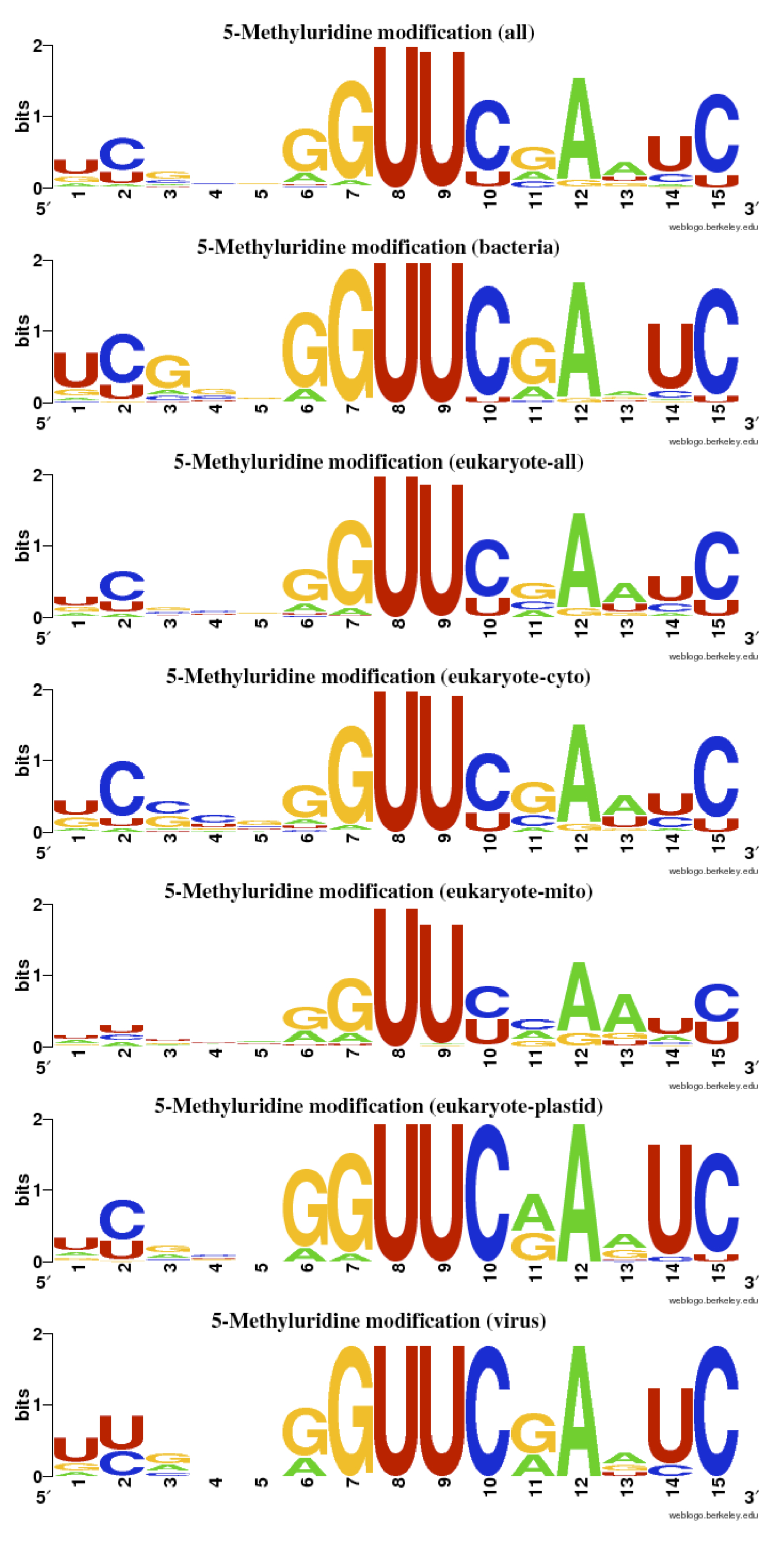
**

**Figure S10: Kingdom-wise WebLogos of 5-methyl-uridine using** **15-length sliding window patterns (central 8^th^ position for 5-methyl-uridine).**

**
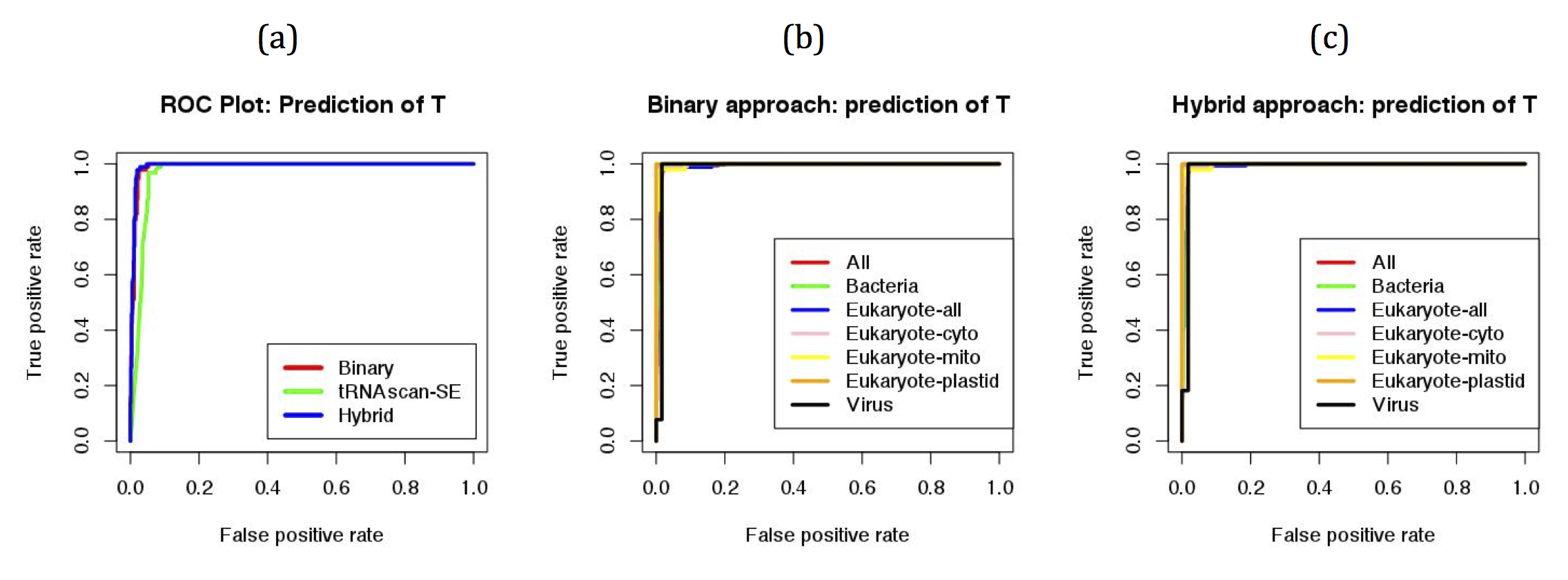
**

**Figure S11: ROC plots showing performances for the prediction of 5-methyl-uridine (T) on the (a) tRNA-136 dataset, (b) BPP appraoch of tRNA-471 dataset and (c) hybrid approach of tRNA-419 dataset.**

**
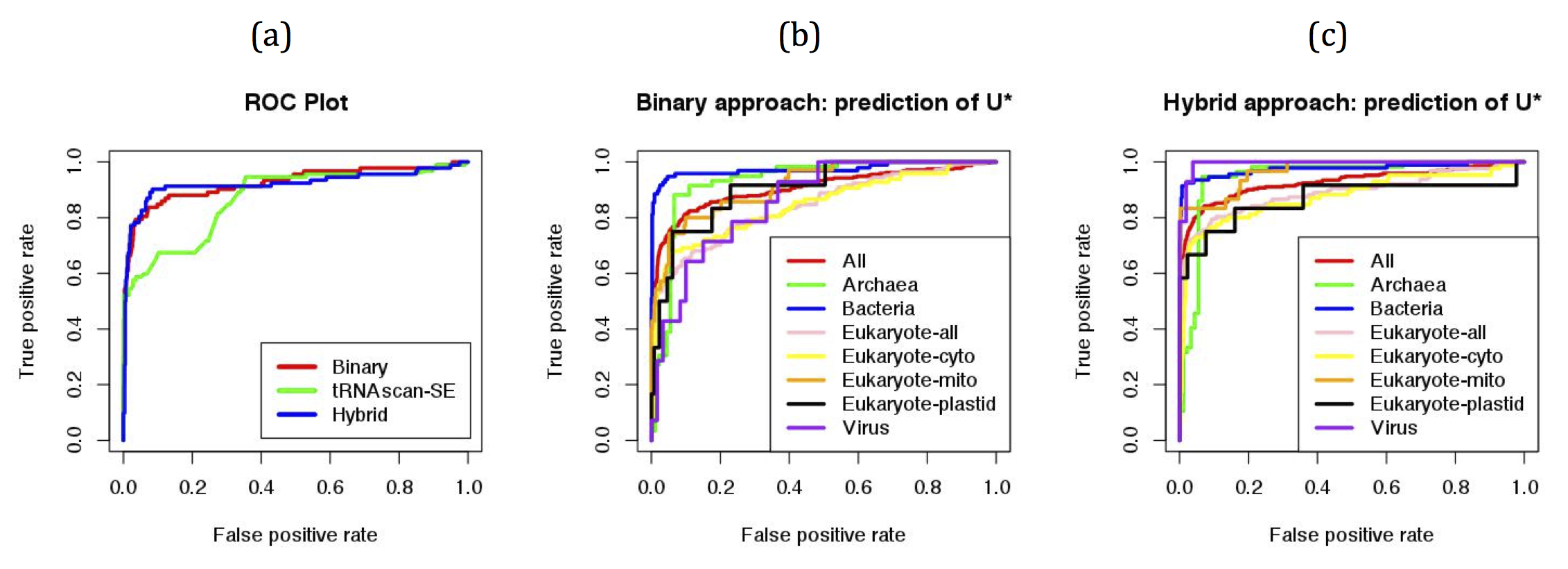
**

**Figure S12: ROC plots showing performances for the prediction of other uridines (except Y, D and T) modifications on the (a) tRNA-136 dataset, (b) BPP appraoch of tRNA-471 dataset and (c) hybrid approach of tRNA-419 dataset.**

**(B) Supplementary Tables**

**Table S1: Modification-wise distribution of 642 tRNAs of the MODOMICS database. The nomenclature of modification used from MODOMICS database.**

| **S.No.** | **No. of modified base** | **Name of modification** | **Original base** |
| --- | --- | --- | --- |
| **1** | **1590** | **P (Y;Pseudouridine)** | **U** |
| **2** | **1129** | **D (Dihydrouridine)** | **U** |
| **3** | **377** | **T (5-methyluridine)** | **U** |
| 4 | 356 | ? | C |
| 5 | 333 | " | A |
| 6 | 267 | L | G |
| 7 | 247 | K | G |
| 8 | 235 | 7 | G |
| 9 | 210 | R | G |
| 10 | 187 | # | G |
| 11 | 172 | B | C |
| 12 | 162 | 6 | A |
| 13 | 109 | N | U |
| 14 | 77 | J | U |
| 15 | 64 | M | C |
| 16 | 60 | . | N |
| 17 | 57 | 4 | U |
| 18 | 56 | ] | U |
| 19 | 54 | I | A |
| 20 | 51 | X | U |
| 21 | 49 | + | A |
| 22 | 46 | O | A |
| 23 | 45 | H | A |
| 24 | 37 | _ | Insertion |
| 25 | 36 | ' | C |
| 26 | 30 | * | A |
| 27 | 28 | ( | G |
| 28 | 25 | Q | G |
| 29 | 24 | = | A |
| 30 | 20 | ; | G |
| 31 | 18 | ! | U |
| 32 | 16 | < | C |
| 33 | 13 | \ | N |
| 34 | 13 | / | A |
| 35 | 12 | Z | U |
| 36 | 11 | W | G |
| 37 | 9 | Y | G |
| 38 | 8 | 3 | U |
| 39 | 8 | E | A |
| 40 | 7 | ) | U |
| 41 | 6 | 1 | U |
| 42 | 6 | 5 | U |
| 43 | 6 | F | U |
| 44 | 6 | V | U |
| 45 | 5 | % | C |
| 46 | 5 | & | U |
| 47 | 5 | ` | A |
| 48 | 4 | 8 | G |
| 49 | 4 | $ | U |
| 50 | 4 | S | U |
| 51 | 4 | ^ | A |
| 52 | 3 | 9 | G |
| 53 | 3 | } | C |
| 54 | 2 | { | U |
| 55 | 2 | > | C |
| 56 | 2 | [ | A |
| 57 | 1 | , | U |
| 58 | 1 | 2 | U |
| 59 | 1 | ° | C |
| 60 | 1 | Â | N |
